# Follow-Up of Patients Diagnosed With Germinal Testicular Tumors (Seminomas and Non-seminomatous) Treated With a Bone Marrow Transplant and a High Dose of Chemotherapy

**DOI:** 10.7759/cureus.61887

**Published:** 2024-06-07

**Authors:** Martin Zapata Laguado, Fernando Contreras Mejia, Mario Pereira Garzon

**Affiliations:** 1 Clinical Oncology, Universidad El Bosque, Bogota, COL; 2 Oncology, Instituto Nacional de Cancerología, Bogota, COL; 3 Hematology, Instituto Nacional de Cancerología, Bogota, COL

**Keywords:** induction chemotherapy, bone marrow transplantation, drug therapy, seminoma, testicular germ cell tumor, testicular neoplasms, non-seminomatous germ cell tumor

## Abstract

Introduction: Germinal testicular tumors are the most common malignant neoplasm in men around 20 to 34 years. Even though they are unusual, they have increased incidence in the last decade; they have an excellent prognosis and overall survival at five years, approximately 95%. Divergent data exists regarding treatment options in patients with first, second, and third relapses with conventional therapy. Some studies describe the possible benefit of using high-dose chemotherapy associated with a bone marrow transplant with variable results.

Methods: The present study describes clinical outcomes, clinical response, mortality, overall survival, and progression-free survival to two years in a group of patients with germinal malignant tumors, seminoma versus non-seminomatous with evidence of progression of the disease at first, second, or third conventional chemotherapy regimens, and who received high dose chemotherapy and bone marrow transplantation at the National Cancer Institute between 2010 and 2021.

Results: A retrospective observational study of case series showed that 57% of patients in third-line therapy received high-dose chemotherapy and bone marrow transplantation, with progression disease median time from diagnosis more than two years. Patients in the post-graft period presented infectious complications (71%). The most common were febrile neutropenia (29%) with a mortality rate of 71% (n=5), progression-free survival of 2.3 months, and overall survival of 7.4 months.

Conclusions: These results show that in this group of patients, regimens with high-dose chemotherapy associated with bone marrow transplants, have a worse prognosis compared to other cohorts of patients, and may not be the best candidates for this rescue therapy.

## Introduction

The most common malignant neoplasia in men between 20 and 34 years is germinal malignancies [1]. Even though they are unusual, they have increased incidence in the last decade; they have an excellent prognosis and overall survival at five years of approximately 95% [2]. Divergent data exists regarding treatment options for patients with first, second, and third relapses with conventional therapy. Some studies describe the possible benefit of using high-dose chemotherapy associated with a bone marrow transplant with variable results [3]. These represent a model of curable neoplasia with platinum-based chemotherapy due to the great sensitivity or response [4]. Treatment options during primary diagnosis comprise evaluation of tumor extension, dissemination to other sites, and tumor markers since 10% to 50% of patients would fail first-line treatment, making it necessary for rescue therapy [2]. The retrospective analysis of 1594 patients performed by the International Prognostic Factors Study Group (IPFSG) identified six clinical, biochemical, and pathological risk factors that allow the oncologist to stratify patients who fail first-line treatment. It stratifies patients into five risk groups and predicts overall survival for three years varying from 6% to 77% [5].

Testicular germinal malignant neoplasms have an excellent prognosis most of the time; however, there is still an area of research for patients with relapse and treatment refractoriness. Standard chemotherapy is associated with good overall survival in patients with germinal malignant neoplasms. Despite the promising results, a subgroup of patients with disease relapse could benefit from high-dose chemotherapy regimens [6]. The literature describes some benefits of high-dose chemotherapy and bone marrow transplantation, especially in subjects whose prognostic features are considered unfavorable. Rescue therapy after first, second, and third-line treatment includes, chemotherapy with backbone of cisplatin in combination with other agents (paclitaxel, vinblastin, ifosfamide, vincristine) or high-dose chemotherapy with carboplatin and etoposide plus autologous transplant of hematopoietic stem cells [7]. Pico et al. in 2005 performed a clinical trial including 280 patients to receive rescue chemotherapy with four cycles of vinblastine or etoposide plus ifosfamide, cisplatin, or third cycles followed by one cycle of carboplatin etoposide and cyclophosphamide. Results published in this cohort found that high-dose chemotherapy did not impact these patients in terms of mortality [8].

Patients with tumors that relapse or progress despite chemotherapy are candidates for rescue chemotherapy, and patients with anatomically localized disease are candidates for rescue surgery plus adjuvant chemotherapy to reduce the probability of relapsing [9]. Cisplatin-containing chemotherapy regimens reach a cure rate of up to 70% in patients diagnosed with metastatic germinal cell malignancies and non-exposed to chemotherapy. It is a well-known prognostic characteristic based on more than 5,000 subjects' studies in different trials, but factors associated with prolonged survival after rescue therapy are less identified [3].

Autologous bone marrow transplant as a rescue therapy after high-dose chemotherapy is a widely known indication in a second-salvage or later setting. However, since 1996, its use has included the administration during the second cycle of high-dose chemotherapy, allowing more time to consolidate and reach complete treatment [3]. Data supporting this type of intervention comes from a retrospective revision of 173 patients with metastatic testicular cancer who presented with disease progression after chemotherapy. This study had a group with an intervention receiving two cycles of high-dose chemotherapy composed of carboplatin plus etoposide, and another group (n=11) receiving a single dose. Results published included 116 patients reaching complete response without relapse and a median follow-up of 48 months. A total of (n=135) patients exposed to high-dose chemotherapy were free of disease in the second-line setting (n=94), and in the third-line setting, 22 of 49 patients were free from the disease at the moment of the last follow-up [3].

Nowadays, an active clinical phase III trial, called the TIGER trial, randomized patients to a reduced conventional dose of chemotherapy vs. high dose chemotherapy plus autologous transplant of bone marrow in the refractory and relapse scenario, as a novel therapeutic strategy in the treatment of germinal cell malignant tumor [10]. Since published clinical trials have demonstrated a clinical benefit derived from bone marrow transplantation associated with a high dose of chemotherapy (progression-free survival of 90% to 24 months and 63% to 48 months, and 30% until 55 months of follow-up), the primary purpose of this original work is to evaluate the impact of this intervention and the clinical outcomes derived in patients at the National Cancer Institute (Instituto Nacional de Cancerología) in Bogota, Colombia.

## Materials and methods

National Cancer Institute's medical records from 2010 to 2021 were reviewed to find patients diagnosed with germinal malignant tumors (seminoma vs. non-seminomatous) treated with high-dose chemotherapy and bone marrow transplant. A non-probabilistic convenience sample allowed us to find seven patients since the implementation of this therapy at the National Cancer Institute was relatively new, and the study was an observational case series study.

Inclusion criteria

Patients diagnosed with a germinal malignant testicular tumor (seminoma and non-seminomatous), without age limitation. Treatment with high-dose chemotherapy and bone marrow transplantation. Medical records from the National Institute of Cancer in Bogota, Colombia, from 2010 to 2021.

Exclusion criteria

Incomplete data from the database. Patients whose information of the total treatment was incomplete. Lack of follow-up during the intervention. The absence of information in medical records about outcome variables such as overall survival and progression-free survival. No precise diagnosis has been confirmed. Patients treated outside the National Cancer Institute. Patients treated with radiation therapy.

Quality assurance and quality control

As an institutional research project, approbation before collecting data from the National Cancer Institute's ethics and research committee was obtained. During the protocol (pre-study, implementation, and closure), support from the institutional monitoring department on research trials assures quality standards.

Data analysis plan

Descriptive analysis derived from the study population allows us to represent frequencies and distributions. The results of the analysis of qualitative variables in distributions by frequencies and percentages and the quantitative variables in means, median, and dispersion were presented in tables. Survival analysis was performed through non-parametric Kaplan-Meier plots. The software used for the analysis was R-Project version 3.6.3 (free license; R Foundation, Vienna, Austria).

Ethical aspects

All the procedures performed during this study were based strictly on international normativity (particularly on the Helsinki Declaration and the ethical guidelines for Biomedical Investigations prepared by the International Council Organization of Medical Science (CIOMS)).

The parameters established in the national scope by "Resolution 8430 of 1993" and "Resolution 2378 of 2008" (expedited by the social protection minister) established the scientific, technical, and administrative rules for investigation in health and issues related to research in human beings. This study considered that research on human subjects should always respect the dignity and veil to protect the rights and well-being of participants.

We considered this research without risk of biological, physiological, psychological, or social variables to the participating subjects. We declare non-conflict of interest of the investigators.

## Results

About 100% of the participants were men, all with testicular non-seminomatous, with good performance status (initial Eastern Cooperative Oncology Group (ECOG)) of 0 and 1, with a median age of 25 years, and a range from 19 to 33 years. About 57% of the participants presented a stage International Germ Cell Cancer Collaborative Group (IGCCCG) initial correspondent to poor risk. The other demographic and clinical features are presented in Table 1.

**Table 1:** Demographic and clinical features (n=7) IGCCCG: International Germ Cell Cancer Collaborative Group; ECOG: Eastern Cooperative Oncology Group

Features	Statistical
	Median (min-max)
Age	25 (19-33)
Corporal weight	71 (39-97)
	n (%)
Sex	
Masculine	7 (100)
Performance status at diagnosis (initial ECOG)	
0	5 (71.43)
1	2 (28.57)
Initial tumoral size	
T0	1 (14.29)
T1	2 (28.57)
T1B	2 (28.57)
T2	1 (14.29)
TX	1 (14.29)
Initial node compromise	
N0	2 (28.57)
N2	1 (14.29)
N3	2 (28.57)
Nx	2 (28.57)
Distant metastases at an initial stage	
M0	1 (14.29)
M1	1 (14.29)
M1a	2 (28.57)
M1b	3 (42.86)
Clinical stage of the disease	
IIC	1 (14.29)
IIIB	3 (42.86)
IIIC	2 (28.57)
IS	1 (14.29)
Initial risk IGCCCG	
Intermedium risk	3 (42.86)
Poor risk	4 (57.14)
Histological type	
Non seminomatous	7 (100)

About 71% of the patients were exposed to orchiectomy as an initial management, of the remaining 29%, one had mediastinal involvement, and the other had retroperitoneal involvement as the primary lesion, with no documentation of a primary testicular lesion. Nearly 71% of the patients received treatment as first-line management with BEP and 57% of the population received second-line management with VeIP. Also, in 57% of the patients, the treatment with high-dose chemotherapy and bone marrow transplantation corresponded to the third line of treatment.

For its part, 57% had a time to progression from the initial treatment of ≥2 years. The other features related to treatment are presented in Table 2.

**Table 2:** Features of the medical treatment BEP: Bleomycin, etoposide y cisplatin; VIP: Etoposide, ifosfamide y cisplatin; VeIP: Vinblastin, ifosfamide y cisplatin

Features	Statistical
	(%)
Initial management	
Orchiectomy	71.43
Other	28.57
First line with chemotherapy	
BEP	71.43
VIP	14.29
Other	14.29
Second line treatment with chemotherapy	
VeIP	57.14
Other	42.86
Third line with chemotherapy	
Gemcitabine, paclitaxel y oxaliplatin	14.29
High-dose chemotherapy and bone marrow transplantation	57.14
Other	28.57
Number of regimens of chemotherapy previous	
2	57.14
≥3	42.86
Sensitivity to platins	
Refractory	14.29
Sensible	85.71
Time to progression from the initial treatment	
<2 years	42.86
≥2 years	57.14
Time to initiate treatment	
0 to 30 days	100
Stage of the disease before transplant	
IIIB	42.86
IIIC	57.14
Status of the disease at the moment of the bone marrow transplantation	
Second partial response or complete	57.14
Third partial response, complete	42.86
Count of stem cells CD34 = infunded	
≥2 x 106 cells/ of weight	100
Movilization scheme	
Other	57.14
PLERIXAFOR + G-CSF or FILGRASTIM 400 mcg AM and 300 mcg PM subcutaneous (Day 1 to 4) - Day 5: 700 mcg total. Before the apheresis of the pH (repeating until reach the target of the recollection of pH)	42.86
Conditioning scheme	
Carboplatin-Etoposide	85.71
Other	14.29
Implanted graft	
Both	57.14
Granulocytic graft	42.86
Number of grafts required	
1	42.86
≥2	57.14

Regarding the tumoral markers related to disease, the results obtained do not show a uniform pattern according to the presented outcomes or to the response initial or post-treatment with a high dose of chemotherapy and bone marrow transplantation, as can be seen in Table 3.

**Table 3:** Biochemical values of follow-up during treatment beta-hCG: beta human chorionic gonadotropin; AFP: Alpha-fetoprotein; LDH: Lactic dehydrogenase

Features	Statistical
	n (%)
beta-hCG	
Initial value	
<1000 IU/liter	3 (42.86)
≥1000 IU/liter	4 (57.14)
Relapse value	
<1000 IU/liter	6 (85.71)
≥1000 IU/liter	1 (14.79)
Follow-up value	
<1000 IU/liter	4 (57.14)
≥1000 IU/liter	3 (42.86)
AFP	
Initial value	
<1000 μg/liter	4 (57.14)
≥1000 μg/liter	3 (42.86)
Relapse value	
<1000 μg/liter	4 (57.14)
≥1000 μg/liter	3 (42.86)
Follow-up value	
<1000 μg/liter	3 (42.86)
≥1000 μg/liter	4 (57.14)
LDH	
Initial value	
<246 U/liter	3 (42.86)
≥247 U/liter	4 (57.14)
Relapse value	
<246 U/liter	4 (57.14)
≥247 U/liter	3 (42.86)
Follow-up value	
<246 U/liter	3 (42.86)
≥247 U/liter	4 (57.14)

**Figure 1:**
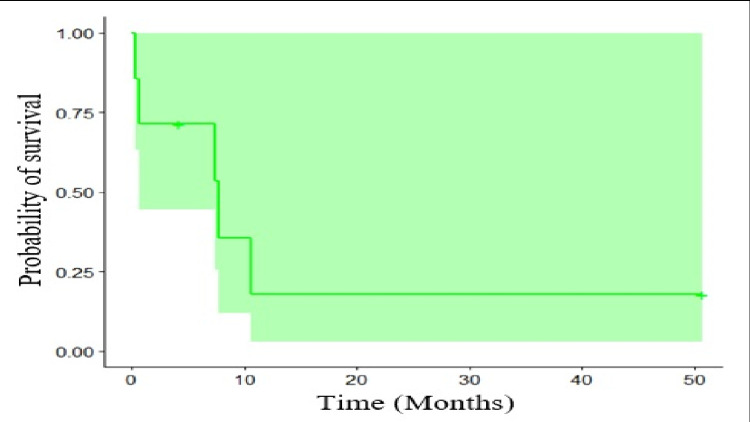
Kaplan-Meier overall survival

In the clinical outcomes presented in the patients, Table 4 showed that 71% presented post-graft infectious complications, being the most frequent febrile neutropenia at 29%. In addition, the prevalence of mortality was 71% with a progression-free survival of 2.3 months and an overall survival of 7.4 months (Figure 1).

**Table 4:** Outcomes related with treatment SOS: hepatic sinusoidal obstruction syndrome

Features	Statistical
	Median (min-max)
Outcomes in graft (months)	1 (0-5)
Disease-free survival (months)	2.3 (1.93-5.53)
Progression-free survival (months)	2.3 (1.93-5.53)
Overall survival (months)	7.4 (0.26-50.66)
	n (%)
Infectious complications of the graft	
Post graft	5 (71.43)
Pre graft	1 (14.29)
Not assessable due to early mortality	1 (14.29)
Toxicity after the graft	
Kidney injury	1 (14.29)
Kidney injury + febrile neutropenia	1 (14.29)
Febrile neutropenia	2 (28.57)
Febrile neutropenia + hepatotoxicity	1 (14.29)
SOS	1 (14.29)
Not assessable due to early mortality	1 (14.29)
Answer 100 days after the treatment proposed	
Stable disease	1 (14.29)
Not assessable due to early mortality	2 (28.57)
Progression of the disease	3 (42.86)
Complete remission (with or without tumoral resection)	1 (14.29)
Immunologic reconstitution	
<30 days	7 (100)
Local or distance recurrence	
No	2 (28.57)
Yes	3 (42.86)
Not assessable due to early mortality	2 (28.57)
Progression, n (%)	
No	2 (28.57)
Yes	3 (42.86)
Not assessable due to early mortality	2 (28.57)
Mortality, n (%)	
No	2 (28.57)
Si	5 (71.43)

## Discussion

High-dose chemotherapy associated with bone marrow transplantation has successfully been used to treat malignant germinal cell relapsing or resistant tumors since the 80s. However, the initial rates of lasting response are approximately 25%, with a mortality rate near 25% [7]. Particularly in this document, we found a prevalence of mortality of 71.43% (n=5) compared with other publications that demonstrate the potential cure for testicular tumors with high-dose chemotherapy plus bone marrow transplantation, even those resistant to platins or used as a third-line or more [7]. For this present study, treatment corresponded to the third line in 57% of the patients.

Feldman et al. published a study with 184 patients whose follow-up reached up to four years with an overall survival of 63% and mortality related of 1.6% [7]. Favorable results are comparable with those found in other studies, in which the disease-free survival oscillates from 20% to 50% [11]. Additionally, in the last years, some adjustments have been made in this field regarding the possibility of receiving treatment in centers with high experience, intensive support, and intensive vigilance of immunological reconstitution after bone marrow transplant. Those facts have improved clinical outcomes in patients, even mortality related to treatment [7].

High-dose chemotherapy and bone marrow transplantation could be helpful as a rescue measure, especially in patients with predictive characteristics, like those with primary testicular tumors that relapse two years posterior to achieve a complete response or partial response to treatment [12]. Our results showed that patients with that type of diagnosis could have time to progress from the initial management of more than two years, and a system score as the one proposed by Einhorn et al. identifies the prognosis of patients managed with high-dose chemotherapy and bone marrow transplantation [3]. This score includes variables such as treatment with third-line or more before deciding on high-dose chemotherapy and bone marrow transplantation, disease refractoriness to platins, and high-risk disease at the moment diagnosis according to the system IGCCCG. Additionally, the score categorizes patients into groups of low-risk, intermediate-risk, and high-risk, with overall survival rates after treatment of approximately 80%, 60%, and 40%, respectively. In addition, it is a helpful tool that could predict results for patients who are candidates for high-dose chemotherapy and bone marrow transplantation [7]. Despite the evidence of this intervention, we found progression-free survival of 2.3 months and overall survival of 7.4 months.

Studies on relapsing malignant testicular germinal tumors treated with chemotherapy with cisplatin could benefit from high-dose chemotherapy. Regimens published include high doses of carboplatin (700 mg/m^2^) and etoposide (750 mg/m^2^) during three consecutive days and followed by an infusion of autologous hematopoietic stem cells of peripheral blood. Results on the strategy exposed above showed favorable results over complete remission of the disease without relapse during a median follow-up of 48 months (rank: 14 to 118) [3]. The cohort of Feldman et al. (n=184) found disease-free survival during follow-up in 51% of the patients who received the intervention mentioned above as second-line rescue therapy, and up to 50% disease-free survival in those who received it as a third-line or posterior. Regarding progression classified by histology type, 60% had seminomas, and 74% had non-seminomatous tumors without evidence of relapse at the last follow-up time [7]. Adverse events reported related to high-dose chemotherapy and bone marrow transplantation were acute leukemia (1.6%), and post-graft infections (71%), with febrile neutropenia being the most common (29%).

High-dose chemotherapy and bone marrow transplantation exist as options for patients with refractory disease in the second and third lines of chemotherapy. Alternative palliative regimens for patients resistant to platins include using gemcitabine with paclitaxel, oxaliplatin, and/or oral etoposide. The use of gemcitabine or oxaliplatin as rescue therapy in patients with non-seminomatous tumor resistance (n=18) in a phase II trial found one subject reaching complete remission and two subjects with a partial response in whom embrionary carcinoma was confirmed histologically [13]. Global response rates in those subjects (n=35) treated previously with platin was 46% [14]. Regimens composed of gemcitabine/oxaliplatin (GEMOX) in 29 patients with non-seminomatous tumors refractory to platins found nine (n=9) patients achieving complete response or partial responses [15].

A retrospective analysis of n=31 subjects found 10 patients reaching partial and 6 complete responses [16]. The combination of gemcitabine, oxaliplatin, and paclitaxel was evaluated in a trial of 63 patients with malignant germinal tumor refractory to platin therapy, with a global response rate of 44% with eight complete remissions in eight patients (13%), and overall survival of 13.3 months. For patients with disease relapse or resistance to treatment, the recommendation is a referral to experienced centers [17].

Nowadays, the first rescue treatment with curative intention after a first-line comprises cycles of chemotherapy with the backbone of platin and matching with ifosfamide, etoposide, or vinblastine, or high-dose chemotherapy with carboplatin and etoposide during 2-3 cycles with subsequent bone marrow transplantation [18]. The first trial phase I/II evaluates a high dose of chemotherapy with two cycles of carboplatin and etoposide plus bone marrow transplantation including 33 patients with disease refractory to cisplatin or after the failure of at least two regimens based on cisplatin. About 25% of subjects reached complete response at expenses of substantial mortality related to treatment (21%) [19]. In our study, 71% of the patients were exposed to orchiectomy as initial management. The treatment was with BEP (71%) and second-line VeIP with 57% of patients in the first progression.

In just one clinical trial, IT-94 evaluated the response comparing high-dose chemotherapy to conventional chemotherapy which demonstrated no benefit in administrating high-dose chemotherapy. However, the investigator used just one cycle, while two or three cycles of high-dose chemotherapy are considered necessary to show an optimal benefit and were used widely as a standard regimen. In contrast, many other retrospective studies, including a publication involving 1594 patients, suggest the benefit from progression-free survival and overall survival for high dose and conventional dose as an initial rescue [10]. The TIGER trial (NCT02375204) is an open clinical trial, multicentric, international, randomized, recruiting actively in collaboration with the American Alliance for Clinical Trials in Oncology and the European Organization for the Investigation and Treatment with Cancer (EORTC). Selected sites in Europe, the United States of America, and Australia pretend to recruit participants to evaluate conventional-dose using the regimen TIP vs. high-dose chemotherapy plus bone marrow transplantation in refractory and relapsing disease. The analysis includes the estimation of overall survival, progression-free survival, response rate, reduction of tumoral markers, and clinal parameters related to toxicity and mortality [10].

## Conclusions

Patients with refractoriness to first, second, and third-line treatment for malignant metastatic germinal tumors (seminoma and non-seminomatous) show poor clinical outcomes including overall survival, progression-free survival, and high incidence of complications due to high doses of chemotherapy plus bone marrow transplant including high grade 4 hematologic toxicity, in the population under study, with a grim oncological prognosis despite of the rescue therapy. Actually TIGER trial would solve the remaining unanswered question of high-dose chemotherapy plus bone marrow transplant would be the standard of care option in second-line therapy.
